# Mesenchymal stromal cells plus basiliximab, calcineurin inhibitor as treatment of steroid-resistant acute graft-versus-host disease: a multicenter, randomized, phase 3, open-label trial

**DOI:** 10.1186/s13045-022-01240-4

**Published:** 2022-03-07

**Authors:** Ke Zhao, Ren Lin, Zhiping Fan, Xiaoyong Chen, Yu Wang, Fen Huang, Na Xu, Xi Zhang, Xin Zhang, Li Xuan, Shunqing Wang, Dongjun Lin, Lan Deng, Danian Nie, Jianyu Weng, Yonghua Li, Xiaohui Zhang, Yuhua Li, A. P. Xiang, Qifa Liu

**Affiliations:** 1 Department of Hematology, Nanfang Hospital, Southern Medical University, Guangzhou, 510515 China; 2 Center for Stem Cell Biology and Tissue Engineering, Sun Yat-Sen University, Guangzhou, 510080 China; 3 Department of Hematology, Peking University People’s Hospital, Beijing, 100044 China; 4 Medical Center of Hematology, Xinqiao Hospital, Army Medical University, Chongqing, 400037 China; 5 Department of Hematology, Guangzhou First People’s Hospital, Guangzhou Medical University, Guangzhou, 510180 China; 6 Department of Hematology, The Third Affiliated Hospital of Sun Yat-Sen University, Guangzhou, 510630 China; 7 Department of Hematology, Zhujiang Hospital, Southern Medical University, Guangzhou, 510282 China; 8 Department of Hematology, Sun Yat-Sen Memorial Hospital of Sun Yat-Sen University, Guangzhou, 510120 China; 9 Department of Hematology, Guangdong Provincial People’s Hospital, Guangdong Academy of Medical Sciences, Guangzhou, 510080 China; 10 Department of Hematology, General Hospital of Southern Theatre Command, Guangzhou, 440104 China; 11 Department of Hematology, The Seventh Affiliated Hospital of Sun Yat-Sen University, Shenzhen, China; 12 Department of Hematology, Shanghai Ninth People’s Hospital, Shanghai Jiao Tong University School of Medicine, Shanghai, China

**Keywords:** Mesenchymal stromal cell, Steroid-resistant acute graft-versus-host disease, Second-line treatment, Allogeneic hematopoietic stem cell transplantation

## Abstract

**Background:**

Steroid-resistant (SR) acute graft-versus-host disease (aGVHD) lacks standard second-line treatment. Mesenchymal stromal cells (MSCs) have potential efficacy in SR aGVHD. We aimed to assess the efficacy and safety of MSCs combined with basiliximab and calcineurin inhibitor as second-line therapy for SR aGVHD.

**Methods:**

A randomized phase 3 trial involved 203 SR aGVHD patients at nine centers in China (September 2014–March 2019). Participants were randomized at a 1:1 ratio to receive second-line therapy with (*n* = 101) or without (*n* = 102) MSCs. The primary endpoint was the overall response (OR) at day 28. Secondary and safety endpoints included durable OR at day 56, failure-free survival, overall survival (OS), chronic GVHD (cGVHD), infection, hematological toxicity and relapse.

**Results:**

Of 203 patients, 198 (97.5%; mean age, 30.1 years; 40.4% women) completed the study. The OR at day 28 was higher in the MSC group than the control group (82.8% [82 patients] vs. 70.7% [70]; odds ratio, 2.00; 95% confidence interval [CI], 1.01–3.94; *P* = 0.043). The durable OR at day 56 was also higher in the MSC group (78.8% [78 patients] vs. 64.6% [64]; odds ratio, 2.02; 95% CI, 1.08–3.83; *P* = 0.027). The median failure-free survival was longer in the MSC group compared with control (11.3 months vs. 6.0 months; hazard ratio (HR) 0.68; 95% CI, 0.48–0.95, *P* = 0.024). The 2-year cumulative incidence of cGVHD was 39.5% (95% CI, 29.3–49.4%) and 62.7% (51.4–72.1%) in the MSC and control groups (HR 0.55, 95% CI, 0.36–0.84; *P* = 0.005). Within 180 days after study treatments, the most common grade 3 and 4 adverse events were infections (65 [65.7%] in the MSC group vs. 78 [78.8%] in the control group) and hematological toxicity (37 [37.4%] vs. 53 [53.5%]). The 3-year cumulative incidence of tumor relapse was 10.1% (95% CI, 5.2–17.1) and 13.5% (7.5–21.2%) in the MSC and control groups, respectively (HR 0.75, 95% CI, 0.34–1.67, *P* = 0.610).

**Conclusions:**

MSCs plus second-line treatments increase the efficacy of SR aGVHD, decrease drug toxicity of second-line drugs and cGVHD without increasing relapse, and are well-tolerated. MSCs could be recommended as a second-line treatment option for aGVHD patients.

*Trial registration* clinicaltrials.gov identifier: NCT02241018. Registration date: September 16, 2014, https://clinicaltrials.gov/ct2/show/NCT02241018.

**Supplementary Information:**

The online version contains supplementary material available at 10.1186/s13045-022-01240-4.

## Background

Acute graft-versus-host disease (aGVHD) remains one of the most frequent complications following allogeneic hematopoietic stem cell transplantation (allo-HSCT) with high mortality [1–5]. Corticosteroids are considered the first-line standard treatment for aGVHD; however, the response rate is only approximately 50%, and long-term survival is poor for those with steroid-resistant (SR) aGVHD [6–9]. Currently, standard second-line treatments for SR aGVHD have not been established [8, 10–13]. Available second-line therapy options include mycophenolate mofetil (MMF), anti-CD25 antibody, ruxolitinib, and so on [10, 14–20]. Deaths from SR aGVHD are only partly due to aGVHD itself, but are mostly due to the long-term influences of aGVHD, such as adverse effects of immunosuppressive agents such as infections and relapse, as well as chronic graft-versus-host disease (cGVHD) evolving from aGVHD. Therefore, new therapeutic agents are urgently needed for the management of SR aGVHD.

Mesenchymal stromal cells (MSCs) are multipotent progenitor cells that exist in various adult tissues, including bone marrow (BM) [21–24]. Based on their multipotency and immunomodulatory properties, they have been used successfully in the treatment of tissue repair and autoimmune diseases, including aGVHD [22, 25, 26]. Since 2004, Le Blanc et al. first reported that MSCs successfully rescued a pediatric patient experiencing refractory aGVHD, an increasing number of studies have been performed to investigate the effect of MSCs in aGVHD treatment [27–38]. Most studies, including our previous non-randomized study, suggested that MSCs were effective for SR aGVHD, but some studies showed that MSCs failed to improve the overall response (OR) of SR aGVHD, for example, a recent industrial MSC-led randomized controlled trial (RCT) [30]. Currently, although debates regarding MSCs as a treatment option for aGVHD are still ongoing, MSCs are recommended as evidence level A-II for aGVHD treatment [10]. However, not enough data from well-designed RCTs are available to verify the second-line treatment position of MSCs for aGVHD. In all previous prospective and retrospective studies, drug combinations exhibited considerable heterogeneity that had a strong impact on efficacy evaluation. In this study, we designed a phase 3 RCT to investigate the efficacy and safety of MSCs combined with second-line drugs for SR aGVHD, in which basiliximab and calcineurin inhibitor were “specified standardized second-line therapy.”

## Methods

### Study design and patients

This study was an open-label, multicenter, randomized, prospective, phase 3 trial conducted at nine hospitals in China between September 2014 and March 2019. Patients were eligible if they were aged 14 to 65 years and diagnosed with SR aGVHD [6, 16, 39]. Patients were excluded if aGVHD occurred due to tapering/discontinuing immunosuppressors or donor lymphocyte infusion (DLI) for prevention/treatment of primary disease relapse, received more than one previous treatment for SR aGVHD except for steroids before randomization, had uncontrolled infections, active visceral hemorrhage, or severe concomitant conditions not suitable for the trial. The diagnosis of aGVHD was according to the literature criteria established by the Mount Sinai Acute GVHD International consortium group [40]. SR aGVHD was defined as aGVHD worsening after 3 days of therapy onset with ≥ 2 mg/kg/day of methylprednisolone or equivalent, or failure to improve after 7 days of treatment initiation; or treatment failure during steroid taper (i.e., an increase in the methylprednisolone dose to ≥ 2 mg/kg/day or equivalent or an inability to taper the dose to < 0.5 mg/kg/day of methylprednisolone or equivalent for a minimum of 7 days) [6, 16, 39].

Approval was obtained from the institutional review board of each participating hospital, and all patients (or their guardians) provided written informed consent before enrollment. This study was performed in accordance with the Declaration of Helsinki.

### Randomization and masking

Once evaluated as eligible, patients were randomly allocated to the MSC and control groups at a ratio of 1:1 according to the randomization principle after signing informed consent form. Randomization was performed with permuted blocks (block size four), and implemented through an interactive web-based response system. The statistical vendor generated the randomization codes, which were given to the interactive response system vendor to perform the randomization. Study site staff enrolled patients. The next assignment in the sequence remained concealed, as treatment was assigned remotely. Treatment allocations were not masked to the investigators or participants. The data analysis and assessments of outcomes were performed in a masked manner.

### MSC preparation

MSCs were manufactured and provided by the Center for Stem Cell Biology and Tissue Engineering, Sun Yat-Sen University. MSCs were obtained from fresh BM of unrelated, HLA-mismatched, third-party donors after written informed consent. Isolation, culture and identification of MSCs were performed in accordance with our previous publication [31, 41–43]. Cells were harvested at passages 4 to 5, and fresh meeting release criteria MSCs were shipped to the clinical sites in 100 ml saline with a continuous temperature monitoring device at 4 °C (Additional file 1: Methods S1).

### Interventions

**Fig. 1:**
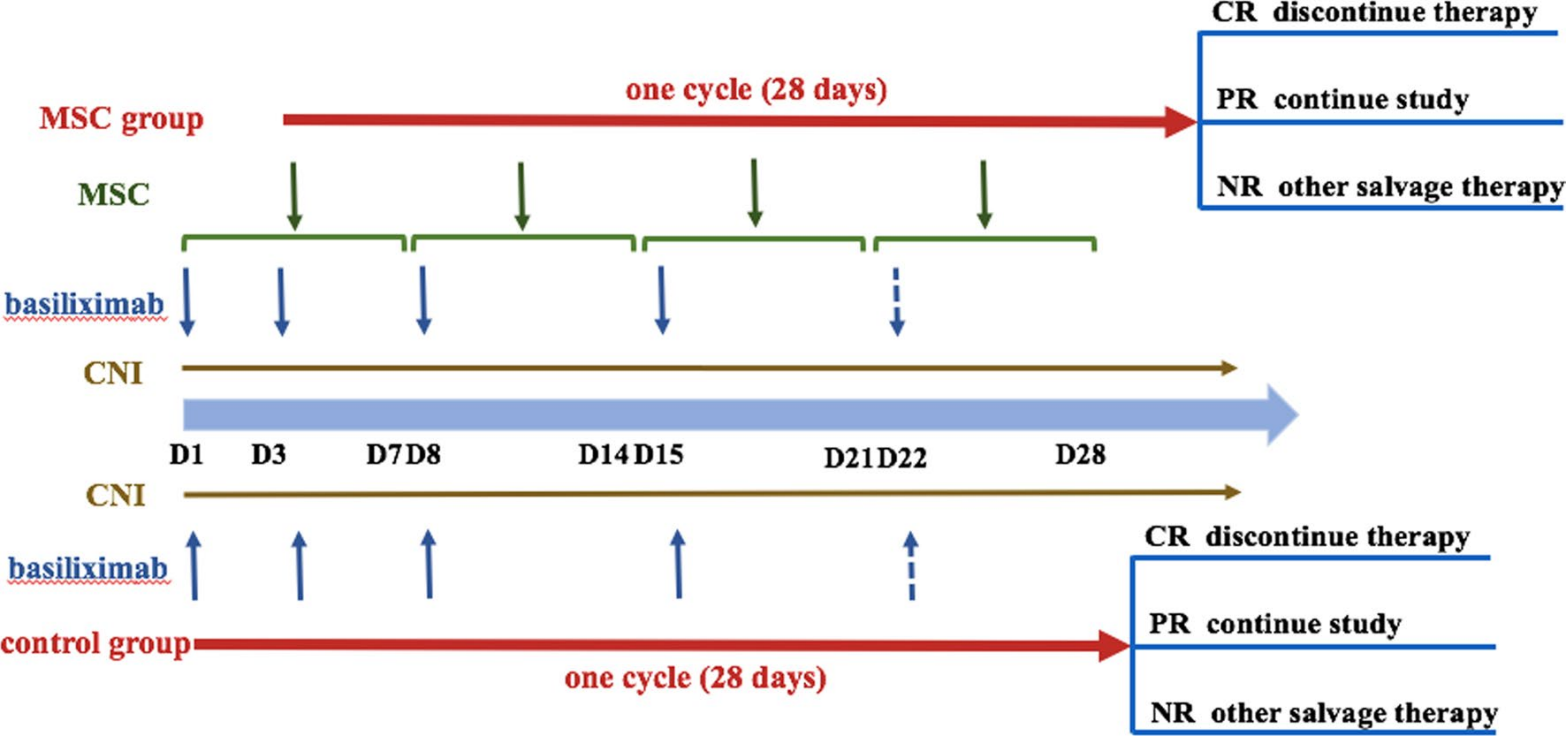
Treatment plan of SR aGVHD patients in the MSC and control group. *CNI* calcineurin inhibitor, *CR* complete response, *PR* partial response, *NR* no response

For patients assigned to the control group, basiliximab (20 mg per dose on day 1, 3, 8, and repeated weekly until aGVHD was reduced to grade < II) and calcineurin inhibitor (first choosing cyclosporine, if not tolerant, change to tacrolimus) considered as “specified standardized second-line therapy” were given in the first cycle (time from the initial treatments to continuous 28 days after that). Steroids were tapered after two doses of basiliximab and recommend tapering by 30% every 5 days and stopping within 4 weeks [44]. Other immunosuppressive agents, such as MMF, methotrexate (MTX), ruxolitinib and mammalian target of rapamycin (mTOR) inhibitor, were allowed after one cycle in NR patients by the attending physician. NR patients evaluated at day 28 in the control group could choose to receive MSCs treatment based on their voluntary principle (Fig. 1).

Patients assigned to MSC group also received “specified standardized second-line therapy” in the first cycle (time from the first dose of MSC infusion to continuous 28 days after that), and other immunosuppressive agents after one cycle in NR patients as the control group. MSCs were initiated within the following 7 days after the application of standardized second-line therapy. MSCs were given intravenously at a dose of 1 × 10^6^ cells/kg once weekly for 4 consecutive weeks as a cycle. Further administration of MSCs was based on the response of MSCs evaluated at day 28. Complete response (CR) and no response (NR) patients discontinued MSCs treatment, while partial response (PR) patients continued to receive MSCs until aGVHD showed CR or MSCs had been infused for 8 doses (Fig. 1).

Patients visited every day from day 1 to day 7, weekly from day 8 to day 56, every month from day 56 to the third month and every 3 months thereafter to collect data on progression, survival, cGVHD and safety outcomes including relapse and infection.

### Endpoints and assessments

The primary endpoint was the OR at day 28, which was defined as the proportion of patients who achieved CR and PR at day 28. The key secondary endpoint was the durable OR at day 56, which was defined as the proportion of patients who had response at day 28 and maintained until day 56. Other secondary endpoints included failure-free survival (time from randomization to relapse or progression of hematologic disease, non-relapse-related death, or the addition of new systemic therapy for aGVHD; the competing risk was the onset of cGVHD) [16], overall survival (OS), the incidence and severity of cGVHD, relapse and non-relapse mortality (NRM). The diagnosis of cGVHD was according to the NIH criteria [45].

Safety analyses were assessed by monitoring adverse events (AEs) and tumor relapse in all patients throughout the trial. AEs included infusion toxicity and infections, hematologic toxicity, et al., which were graded according to the National Cancer Institute Common Terminology Criteria for Adverse Events version 4.0. Trial drug infusion-related safety was assessed by a physician investigator who remained at the patient’s bedside for the duration of the infusion and in intensive care unit for 6 h after the start of infusion to monitor for AEs. Follow-up care was monitored by physical examination and laboratory assessments, such as routine blood testing, liver, renal function and myocardial enzymes, BM assessment, CMV-DNA and EBV-DNA, et al. Grade 3 hematologic AEs were defined as ANC < 1.0–0.5 × 10^9^/L or PLT < 30–20 × 10^9^/L, and grade 4 hematologic AEs as ANC < 0.5 × 10^9^/L or PLT < 20 × 10^9^/L [46].

### Statistical analysis

The sample size was calculated based on the primary endpoint, the OR rate of MSCs treatment for SR aGVHD, which was approximately 70% in a previous study [28]. To identify a 20% difference in OR rate of SR aGVHD with MSCs plus second-line drugs treatment, a minimum of 93 patients per group was required to provide the study with 80% power and a two-sided significance level of 0.05. Considering a dropout rate of 5%, sample size was increased to 98 patients for each group. The sample size calculation was conducted using PASS version 15 software.

Statistical analysis was performed using the intent-to-treat (ITT) population on June 30, 2020. ITT population was defined as all randomly assigned patients, which was the basis for the analysis of efficacy and safety endpoints. The incidence and severity of cGVHD were performed in the modified ITT (mITT) population, which excluded patients who received DLI as a prevention/treatment for relapse and MSCs as a salvage treatment for refractory aGVHD in the control group. All statistical analyses were performed using software SPSS 21.0 or R version 3.3.0. Patient data were compared using Fisher’s exact test for categorical variables and Mann–Whitney U tests for continuous variables. Kaplan–Meier curves for failure-free survival and OS were plotted, and the hazard ratios (HR) were calculated, along with the 95% confidence intervals (CI), with the use of a stratified Cox model. The cumulative incidence of cGVHD, relapse and NRM were calculated by accounting for competing risks. Competing risks for cGVHD included relapse and death without cGVHD. Relapse was a competing risk for NRM, and NRM was a competing risk for relapse. The comparison of the cumulative incidence in the presence of a competing risk was performed using the Fine and Gray method [47]. *P* < 0.05 for a two-sided text was considered statistically significant.

## Results

### Patients

**Fig. 2:**
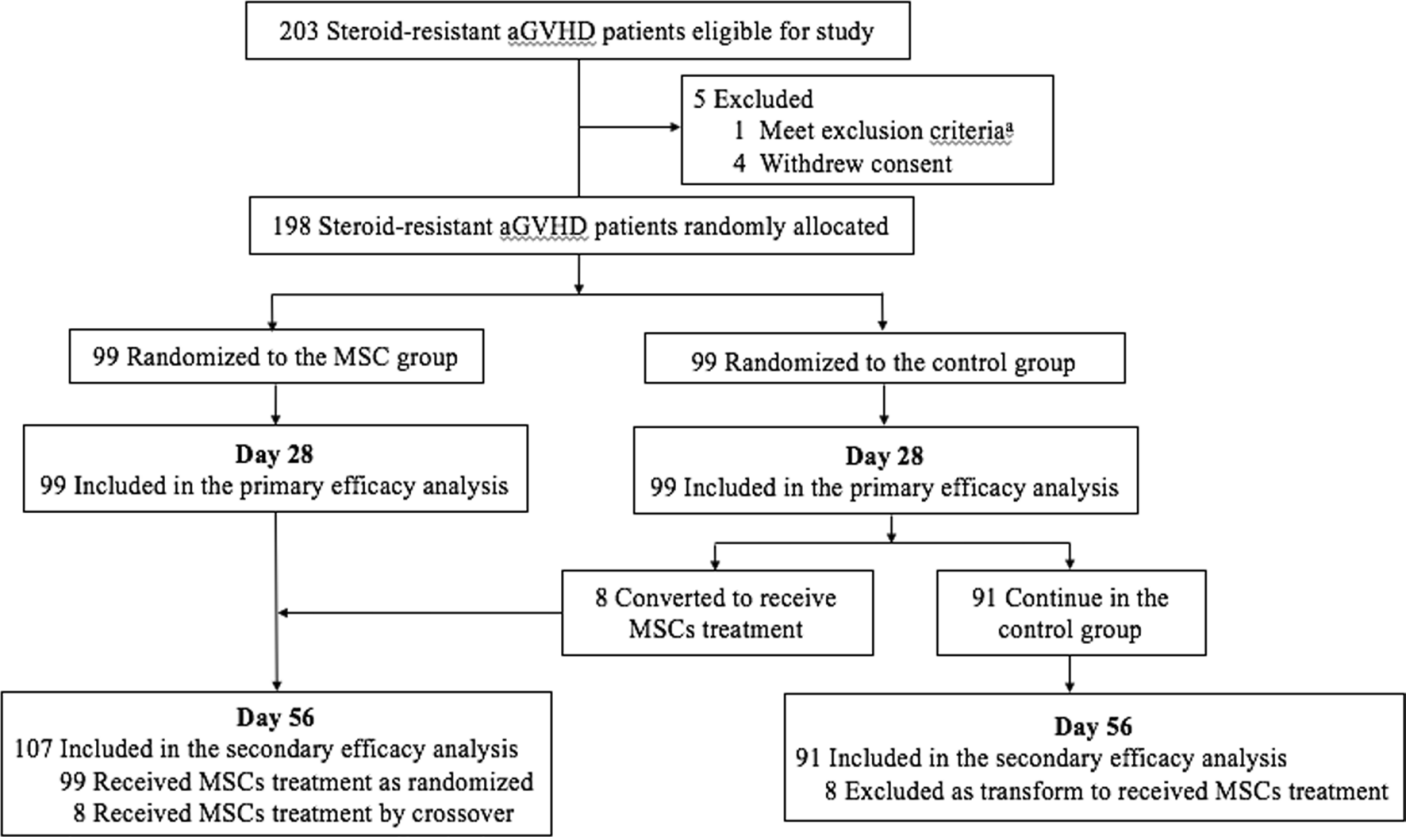
Flow of patient enrollment, randomization and follow-up

Between September 2014 and March 2019, a total of 203 patients with SR aGVHD were screened at enrollment, four of which withdrew informed consent and one met exclusion criteria. The remaining 198 patients were enrolled and randomly assigned to the MSC group (99 patients) or control group (99). The study flow diagram is shown in Fig. 2.

The baseline demographic, GVHD, transplantation-related and disease-related characteristics of patients in two groups are given in Table 1. Of 198 enrolled patients, the median age was 29 years (range, 14–59). A total of 73 patients (36.9%) had grade II aGVHD, 85 (42.9%) had grade III, and 40 (20.2%) had grade IV aGVHD. 19 (9.6%) patients developed upper gastrointestinal (GI) aGVHD, 156 (78.8%) developed lower GI aGVHD, 136 (68.7%) developed skin, and 89 (44.9%) developed liver aGVHD. The median time from transplantation to diagnosis of aGVHD was 30 days (14–132) in the MSC group and 28 days (16–124) in the control group. The two groups were balanced with respect to age, sex, primary disease and disease status at transplant, transplant modality and aGVHD characteristics.

**Table 1:** Baseline, disease, transplantation and GVHD characteristics of patients with SR aGVHD in two groups

Variable	MSC group No. (%)	Control group No. (%)	*P*
No. of patients	99	99	
Age, median (range), years	28 (16–59)	29 (16–57)	0.680
< 18 year	14	17	
≥ 18 year	85	82	
Sex			0.385
Male	62 (62.6%)	56 (56.6%)	
Female	37 (37.4%)	43 (43.4%)	
Disease			0.129
AML	39 (39.4%)	49 (49.5%)	
ALL	45 (45.5%)	43 (43.4%)	
Others*	15 (15.2%)	7 (7.1%)	
MDS	6 (6.1%)	3 (3.0%)	
CML	3 (3.0%)	1 (1.0%)	
MM	1 (1.0%)	0	
NHL	0	1 (1.0%)	
Other acute leukemia	5 (5.1%)	2 (2.0%)	
Disease status at transplant			0.524
CR	63 (63.6%)	70 (70.7%)	
PR	6 (6.1%)	6 (6.1%)	
NR	30 (30.3%)	23 (23.2%)	
HLA typing			1.000
HLA matched	51 (51.5%)	51 (51.5%)	
HLA mismatched	48 (48.5%)	48 (48.5%)	
Conditioning			0.200
Bu-based	51 (51.5%)	42 (42.4%)	
TBI-based	48 (48.5%)	57 (57.6%)	
Donor source			0.567
PBSC	53 (53.5%)	57 (57.6%)	
PBSC + BM	46 (46.5%)	42 (42.4%)	
GVHD prevention			0.886
CsA + MTX or CsA + MTX + MMF	42 (42.4%)	43 (43.4%)	
CsA + MTX + MMF + ATG	57 (57.6%)	56 (56.6%)	
Grade of aGVHD			0.771
Grade 2	36 (36.4%)	37 (37.4%)	
Grade 3	41 (41.4%)	44 (44.4%)	
Grade 4	22 (22.2%)	18 (18.2%)	
No. of aGVHD involved organs			0.589
1	25 (25.3%)	29 (29.3%)	
2	46 (46.5%)	48 (48.5%)	
3	28 (28.3%)	22 (22.2%)	
aGVHD involved organs			0.860
Skin	73 (73.7%)	63 (63.6%)	
Liver	43 (43.4%)	46 (46.5%)	
Upper GI	9 (9.1%)	10 (10.1%)	
Lower GI	79 (79.8%)	77 (77.8%)	
Onset of aGVHD median days (range)	30 (14–132)	28 (16–124)	0.736

*GVHD* graft-versus-host disease, *SR* steroid-resistant, *MSCs* mesenchymal stromal cells, *AML* acute myeloid leukemia, *ALL* acute lymphocyte leukemia, *CR* complete response, *PR* partial response, *NR* no response, *HLA* human leukocyte antigen, *Bu* busulfan, *TBI* total body irradiation, *PBSC* peripheral blood stem cells, *BM* bone marrow, *CsA* cyclosporine, *MTX* methotrexate, *MMF* mycophenolate mofetil, *ATG* antithymocyte globulin, *GI* gastrointestinal

*Others included myelodysplastic syndrome (MDS), chronic myelogenous leukemia (CML), multiple myeloma (MM), non-hodgkin lymphoma (NHL) and other acute leukemia

### Efficacy

**Fig. 3:**
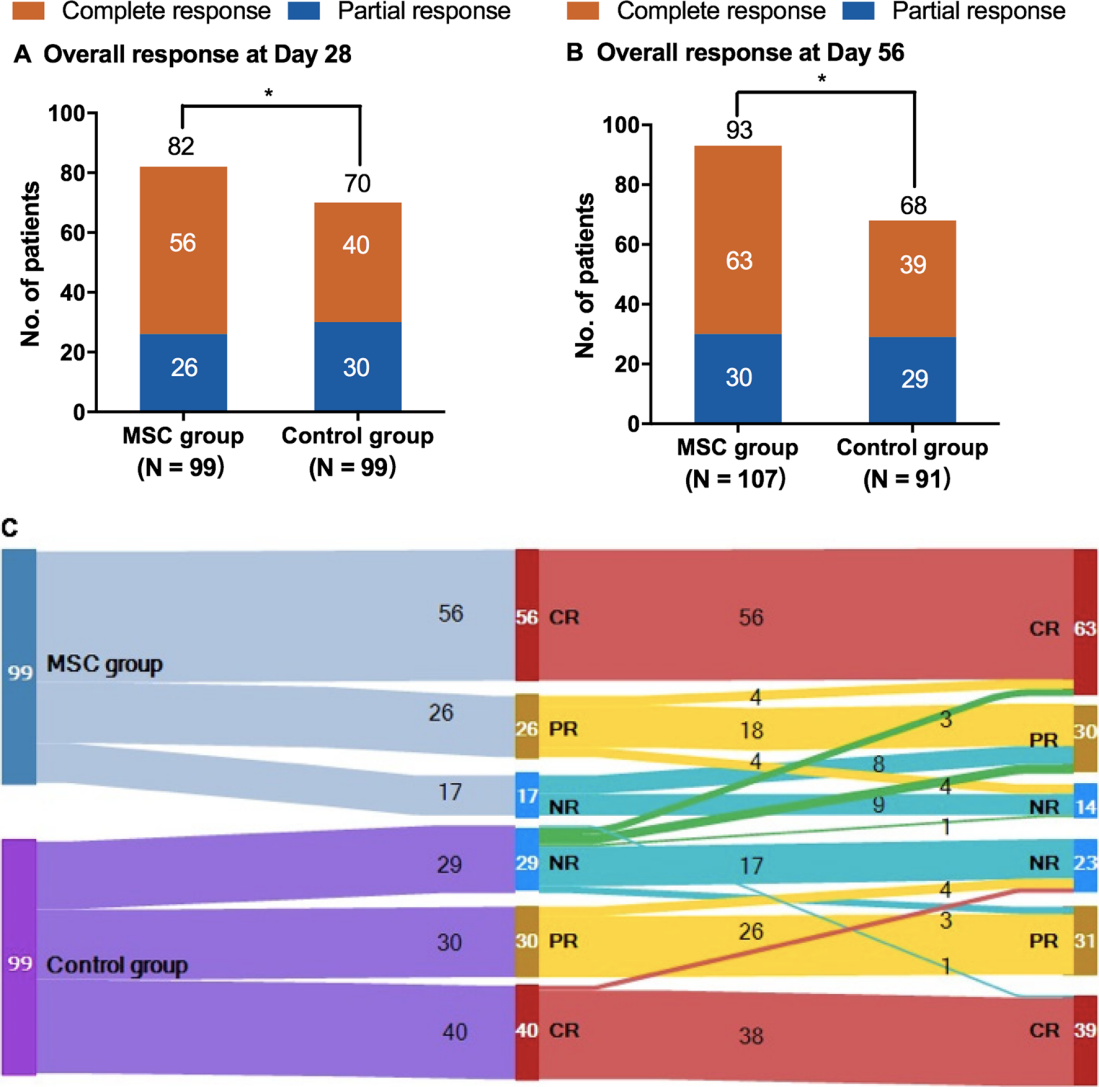
Assessment of response to acute graft-versus-host disease (aGVHD) treatments. **A** Overall response (OR) at day 28 after randomization, **B** OR at day 56 after randomization and **C** Sankey diagram of responses in the MSCs and control groups over time. Steroid-resistant (SR) aGVHD in the MSCs and control groups were shaded baby blue and ultramarine, respectively; the width of each bar represented their relative frequency with the study. Qualities of response at day 28 follow-up (second column from left) and at day 56 follow-up (third column from left) were depicted in red (CR), yellow (PR), and prussian blue (NR). The NR patients in the control group crossed over to receive MSCs treatment was depicted in green

In the MSC group, the median number of MSC infusions for each patient was 5 (3–8). Median duration from the onset of aGVHD to the first MSC infusion was 10 days (6–17). For the primary efficacy evaluation at day 28, 56 of 99 patients (56.6%) achieved CR, 26 (26.3%) achieved PR, and 17 (17.2%) did not respond in the MSC group, while CR in 40 of 99 patients (40.4%), PR in 30 (30.3%) and NR in 29 (29.3%) in the control group. The OR rate at day 28 in the MSC group was significantly higher than that in the control group (82.8% [82 of 99 patients] vs. 70.7% [70 of 99]; odds ratio, 2.00; 95% CI, 1.01–3.94; *P* = 0.043). The proportions of patients with OR were the highest in patients with grade II aGVHD (97.2% [35 of 36 patients] in the MSC group vs. 91.9% [34 of 37] in the control group) and in those with grade III aGVHD (80.5% [33 of 41] vs. 68.2% [30 of 44]). However, the odds ratio for response in the MSC group as compared with control was the highest among patients with grade IV aGVHD (63.6% [14 of 22] vs. 33.3% [6 of 18]; odds ratio, 3.5; 95% CI, 0.95–12.97). The responses of patients with aGVHD in two groups are shown in Table 2 and Fig. 3A–C. The OR rate at day 56 was significantly higher in the MSC group than the control group (86.9% [93] vs. 74.7% [68]; odds ratio, 2.25; 95% CI, 1.08–4.68; *P* = 0.028; Fig. 3A–C). Durable OR at day 56 was also higher in the MSC group (78.8% [78] vs. 64.6% [64]; odds ratio, 2.03; 95% CI, 1.08–3.83; *P* = 0.027).

**Table 2:** Treatment response of SR aGVHD between the two groups at day 28

Outcomes	MSC group	Control group	*P*
No. of patients	99	99	
OR rate of aGVHD grade			
II	35 (97.2%)	34 (91.9%)	0.317
III	33 (80.5%)	30 (68.2%)	0.196
IV	14 (63.6%)	6 (33.3%)	0.057
OR rate of aGVHD involved organs numbers			
1	24 (96.0%)	26 (89.7%)	0.375
》 2	58 (78.4%)	44 (62.9%)	0.002
OR rate of aGVHD organs			
Skin	64 (87.7%)	50 (79.4%)	0.190
Liver	33 (76.7%)	32 (69.6%)	0.446
Upper GI	9 (100.0%)	9 (90.0%)	0.330
Lower GI	54 (68.4%)	46 (59.7%)	0.262
OR rate of patients’ age			
< 18 year	12 (85.7%)	11 (64.7%)	0.183
≥ 18 year	70 (82.4%)	59 (71.9%)	0.109

**Fig. 4:**
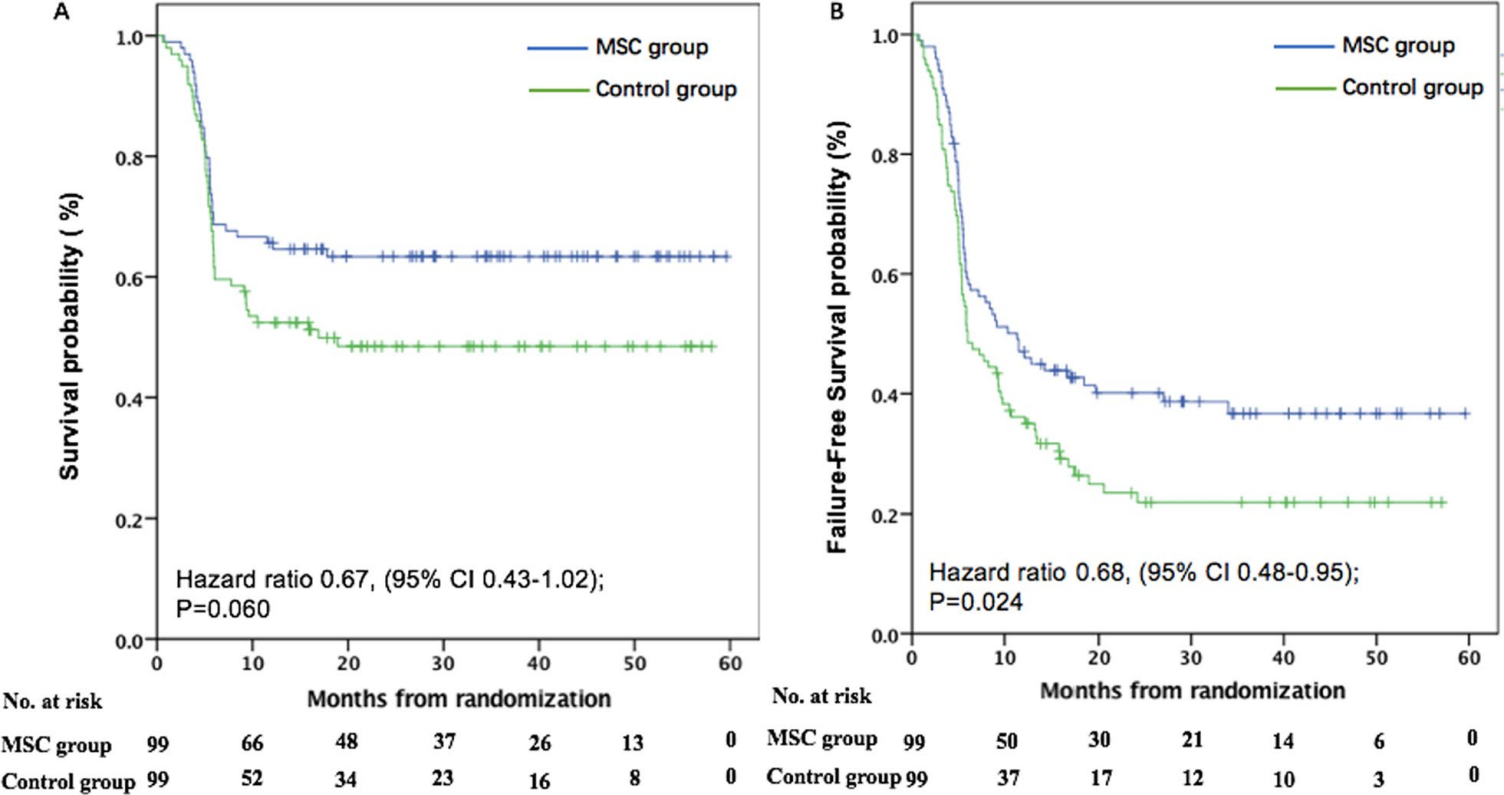
Overall survival (OS) and failure-free survival. **A** OS and **B** failure-free survival were stratified according to whether patients receiving MSCs post-randomization. And for these analysis, the eight patients in the control group who crossed over to receive MSCs are included in the control group. Failure-free survival was defined as time from randomization to relapse or progression of hematologic disease, non-relapse-related death or the addition of new systemic therapy for aGVHD, and the competing risk was the onset of chronic graft-versus-host disease (cGVHD). *P < 0.05, **P < 0.001

The median follow-up was 19.8 months (0.76–59.6) in the MSC group and 12.3 months (0.6–58.1) in the control group. In the MSCs group, 63 patients survived and 36 patients died, while 49 survived and 50 died in the control group. The causes of death in the MSC and control groups included primary disease relapse (*n* = 8 vs. 9), aGVHD (*n* = 9 vs. 14), cGVHD (*n* = 4 vs. 8), severe infections (*n* = 12 vs. 16), hemorrhagic disease (*n* = 3 vs. 2) and thrombotic microangiopathy (*n* = 0 vs. 1). The 6-month, 1-year and 3-year OS were 68.7% (95% CI, 64.0–73.4%), 67.1% (62.3–71.9%) and 63.4% (58.5–68.3%) in the MSC group versus 60.6% (55.7–65.5%), 54.8% (49.7–59.9%) and 48.5% (43.4–53.6%) in the control group, respectively (HR 0.76, 95% CI, 0.47–1.22; *P* = 0.248, HR 0.68, 95% CI, 0.43–1.07; *P* = 0.096, HR 0.67, 95% CI, 0.43–1.02; *P* = 0.060; Fig. 4A). The median failure-free survival was significantly longer in the MSC group than the control group (11.3 months vs. 6.0 months; HR 0.68; 95% CI, 0.48–0.95, *P* = 0.024) (Fig. 4B).

### cGVHD

**Fig. 5:**
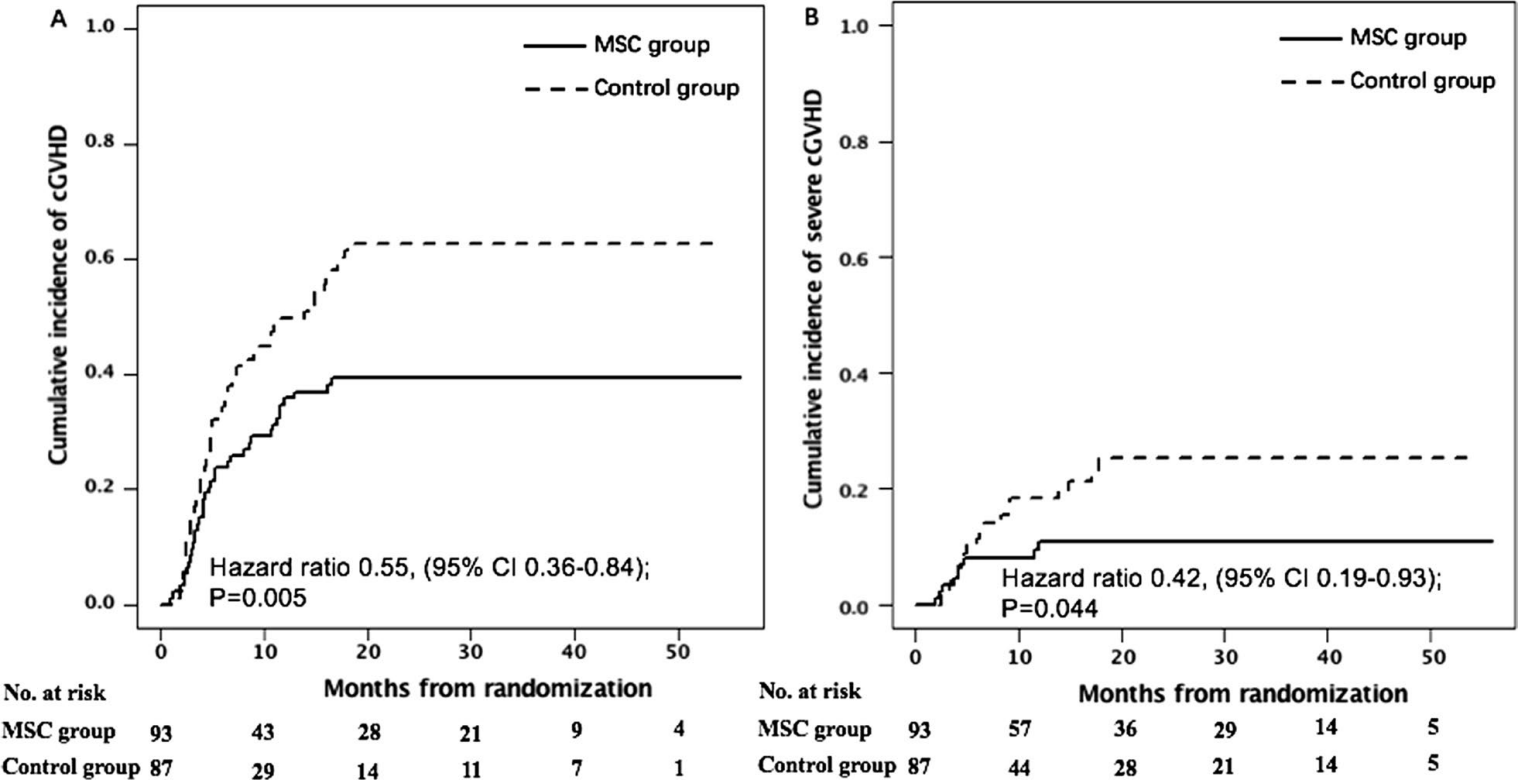
Cumulative incidence of overall chronic graft-versus-host disease (cGVHD) (**A**) and severe cGVHD (**B**). **A**, **B** Stratified according to whether patients receiving MSCs post-randomization. *P < 0.05, **P < 0.001

The 1-year and 2-year cumulative incidence of overall cGVHD was 35.0% (95% CI, 25.3–44.8%) versus 49.7% (38.7–59.8%) (HR 0.57, 95% CI, 0.36–0.91, *P* = 0.046) and 39.5% (29.3–49.4%) versus 62.7% (51.4–72.1%) (HR 0.55, 95% CI, 0.36–0.84, *P* = 0.005; Fig. 5A), and severe cGVHD was 9.4% (4.4–16.9%) versus 18.1% (10.4–27.6%) (HR 0.43, 95% CI, 0.17–1.06, *P* = 0.131) and 10.8% (5.2–18.6%) versus 25.3% (15.6–36.2%) (HR 0.42, 95% CI, 0.19–0.93, *P* = 0.044; Fig. 5B) in the MSC and control groups, respectively.

### Safety

AEs from enrollment to 180 days after study treatments are shown in Table 3. Multiple infusions of MSCs were well-tolerated with no infusion-related AEs during infusion or within 6 h from the start of infusion. At least one type of grade 3–4 AE was reported for 83 (83.8%) of 99 patients in the MSC group and 85 (85.9%) of 99 in the control group. The most common grade 3–4 AEs for patients assigned to the MSC and control groups were infection and hematologic toxicity. Infection of any grade 3–4 occurred in 65 patients (65.7%) who received MSCs and in 78 (78.8%) who received control therapy (*P* = 0.039). Among patients with infection, the viral, bacterial and fungal infections in the MSC group, respectively, accounted for 69.2%, 38.5% and 10.8%, compared with 70.5%, 46.2% and 12.8% in the control group. Grade 3–4 hematologic toxicities occurred in 37 patients (37.4%) in the MSC group and 53 (53.5%) in the control group (*P* = 0.022).

**Table 3:** Adverse events reported by interventional investigators

Event	MSC group (*N* = 99)		Control group (*N* = 99)	
	Any grade	Grade ≧ 3	Any grade	Grade ≧ 3
Any adverse event	92 (92.9)	83 (83.8%)	95 (96.0%)	85 (85.9%)
Hematologic^a^	–	37 (37.4%)	–	53 (53.5%)
Platelets decreased	–	21 (21.2%)	–	30 (30.3%)
Neutrophils decreased	–	16 (16.2%)	–	23 (23.2%)
Skin^b^	27 (27.3%)	8 (8.1%)	32 (32.3%)	12 (12.1%)
Gastrointestinal^b^	30 (30.3%)	5 (5.1%)	37 (37.4%)	8 (8.1%)
Hepatobilinary or pancreatic^b^	16 (16.2%)	5 (5.1%)	18 (18.2%)	4 (4.0%)
Cardiac	39 (39.4%)	13 (13.1%)	41 (41.4%)	16 (16.2%)
Renal or genitourinary	27 (27.3%)	10 (10.1%)	28 (28.3%)	11 (11.1%)
Vascular	18 (18.2%)	7 (7.1%)	19 (19.2%)	10 (10.1%)
Infections	75 (75.8%)	65 (65.7%)	81 (81.8%)	78 (78.8%)
Secondary malignant disease	0 (0%)	0 (0%)	0 (0%)	0 (0%)

Table shows the adverse events that have an incidence of at least 10% in either group. The safety population included all patients who received at least one dose of trial therapy

^a^Included patients with decreases in platelet counts and neutrophil counts

^b^Excluded patients with aGVHD

Serious AEs (SAEs) occurred in 41 patients (41.4%) of MSC group and in 44 (44.4%) of control group (Table 4). Twenty-four patients in the MSC group and 34 in the control group died from SAE. Most deaths were attributed to serious aGVHD (nine patients [9.1%] in the MSC group and 14 [17.2%] in the control group). Other causes of death during the randomized treatment period were infections (8 vs. 12 patients in the MSC and control groups), relapse (5 vs. 6), and hemorrhagic disease (2 vs. 2). These deaths were not related to treatments.

**Table 4:** Serious adverse effects

Event	MSC group (*N* = 99)	Control group (*N* = 99)
Any adverse event	41 (41.4%)	44 (44.4%)
Hematologic^a^	9 (9.1%)	13 (13.1%)
Platelets decreased	6 (6.1%)	8 (8.1%)
Platelet and neutrophil both decreased	3 (3.0%)	5 (5.1%)
Skin^b^	0 (0%)	1 (1.0%)
Gastrointestinal^b^	4 (4.0%)	5 (5.1%)
Hepatobilinary or pancreatic^b^	2 (2.0%)	2 (2.0%)
Cardiac (heart failure)	4 (4.0%)	6 (6.1%)
Renal or genitourinary (cystitis non-infective)	4 (4.0%)	5 (5.1%)
Vascular (thrombotic microangiopathy)	0 (0%)	1 (1.0%)
Infections	12 (12.1%)	16 (16.2%)
Secondary malignant disease	0 (0%)	0 (0%)

^a^Included patients with decreases in platelet counts and neutrophil counts

^b^Excluded patients with aGVHD

**Fig. 6:**
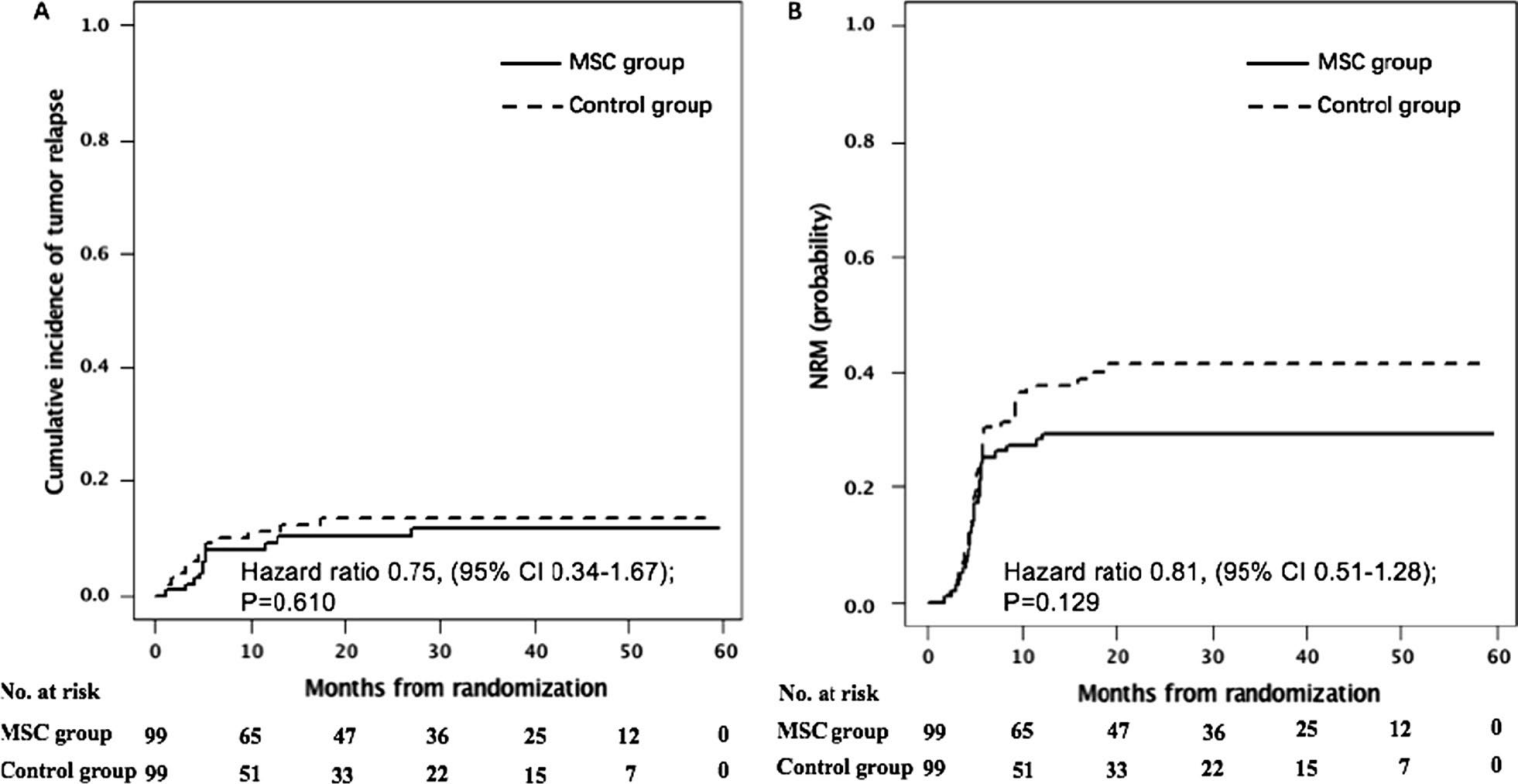
Cumulative incidence of leukemia relapse (**A**) and non-relapse mortality (NRM) (**B**). **A**, **B** Stratified according to whether patients receiving MSCs post-randomization

The 3-year cumulative incidence of relapse was 10.1% (95% CI, 5.2–17.1%) in the MSC group and 13.5% (7.5–21.2%) in the control group (HR 0.75, 95% CI, 0.34–1.67, *P* = 0.610, Fig. 6A). NRM at 3 years was 29.3% (20.6–38.5%) in the MSC group and 41.4% (31.3–51.1%) in the control group (HR 0.81, 95% CI, 0.51–1.28, *P* = 0.129, Fig. 6B).

## Discussion

This open-label, randomized phase 3 trial shows that MSCs plus basiliximab and calcineurin inhibitor for SR aGVHD patients lead to a great improvement in efficacy, with a higher OR at day 28 and higher durable OR at day 56. MSC administration was also associated with prolonged failure-free survival than control. Moreover, we found that MSCs can reduce the side effects of second-line drugs, such as BM toxicity and infections. Distinguished from other studies, we adopted MSCs plus “specified standardized second-line therapy,” which minimized the confounding variable of heterogeneous second-line therapies, so that the results were more comparable.

Currently, we have a wide choice of second-line treatments that could be used to treat SR aGVHD, including ruxolitinib, monoclonal antibodies, MTX, mTOR inhibitor, etc. [10, 14–19]. However, little reliable information to determine which agents might be best for SR aGVHD patients. Therefore, no standard second-line treatments for SR aGVHD have been recommended. Ruxolitinib recently became the first drug approved for SR aGVHD treatment, with high response rates (55–62%) [16]. Anti-CD25 antibody as one of the most commonly used SR aGVHD treatments led to the response of 70.2% [15]. MSCs have been investigated in a large number of clinical trials as novel cellular therapy in GVHD [27–38]. In a phase II single-arm study involving 55 SR aGVHD patients with MSC treatment, OR rate was 70.9% [28]. In our preliminary non-randomized pilot study, we observed that MSCs led to a higher OR than control in aGVHD patients who failed second-line treatment [31]. In this RCT, we focused on SR aGVHD patients treated with MSCs plus basiliximab and calcineurin inhibitor as the “specified standardized second-line therapy”. The results showed that MSCs plus second-line drugs had a better response than treatment of single agent for SR aGVHD. But owing to unbalanced treatment cohorts and different definitions and timing of response assessments, the comparison needed to be caution. In contrast with these results, some studies documented that MSCs failed to improve the low response rate. Recent RCT based on the addition of industrial MSCs to heterogeneous second-line therapies in SR aGVHD patients failed to improve the durable CR at day 28 compared with the control [30]. Among all previous studies, drug combinations exhibited considerable heterogeneity that had a strong impact on efficacy evaluation. Besides, we surprisingly found that the NR SR aGVHD patients who received ruxolitinib in MSC group showed a higher efficacy than the control group (42.8% (3/7) vs. 11.1% (1/9), respectively). But the sample size is too small and large-scale clinical trials are needed.

What accounts for the opposite clinical outcomes concerning the efficiency of MSCs for aGVHD? The heterogeneity of MSC products partly explained the difference, which included MSCs source, manufacturing process, donors, culture passages, and the culture and expansion media [22, 48, 49]. Moreover, the heterogeneity of enrolled patients and treatment schedule also influenced the effects of MSC treatment [22, 28–31, 49]. The highlight of our RCT is that we standardized the second-line therapies for aGVHD in the MSC and control groups. To our knowledge, no RCT has been designed to eliminate the nonstandard influence of second-line drug combinations in MSC efficacy evaluation for aGVHD treatment.

Regarding safety, there remains debates over whether MSCs increase relapse, infection and BM suppression toxicity. Most studies have indicated that MSCs do not increase infection or relapse. However, Ning et al. reported that MSCs increased relapse in patients co-transplanted HSCs to prevent GVHD [50]. This study showed that relapse did not differ between the MSC and control groups. Of interest, we found that infection was improved by MSC treatment. The rational explanations are that MSCs promoting T-cell reconstitution and possessing antimicrobial ability by direct effects on pathogens or indirect effects through secreting soluble factors and enhancing anti-inflammatory function of immune cells [51–56]. Another interesting discovery is that MSCs improve BM toxicity, possibly because MSCs play a vital role in modulating BM microenvironment and supporting hematopoiesis [22, 25, 31, 57].

In addition, we found that the 2-year cumulative incidence of overall cGVHD and severe cGVHD was both decreased in the MSC group compared with controls, verifying our previous explore findings [31]. The mechanisms might be associated with MSCs alleviating thymus damage caused by aGVHD by improving the thymic negative selection, decreasing auto-reactive T-cell and inducing Treg production [31, 58–61].

A few highly relevant shortcomings of data presented here should be mentioned. First, this is a non-blinded and non-placebo controlled study, which may carry a higher risk of bias on the part of both the treating physician and the patient, usually in favor of the investigational arm. Moreover, SR aGVHD in our study was almost always diagnosed by clinical findings, which might influence the therapeutic evaluation of MSCs.

## Conclusions

This trial shows that the addition of BM-derived third-party MSCs to second-line therapy leads to a higher therapeutic response and prolonged failure-free survival of SR aGVHD patients compared with controls. MSCs also decrease toxicity of second-line drugs and cGVHD without increasing relapse. MSCs could be recommended as a second-line treatment option for aGVHD patients.

## Supplementary Information

**Additional file 1: Method S1.** Preparation of Mesenchymal Stromal Cells.

## Data Availability

The datasets used and/or analyzed during the current study are available from the corresponding author on reasonable request.

## Abbreviations

aGVHD: Acute graft-versus-host disease
allo-HSCT: Allogeneic hematopoietic stem cell transplantation
SR: Steroid-resistant
MMF: Mycophenolate mofetil
cGVHD: Chronic graft-versus-host disease
MSCs: Mesenchymal stromal cells
BM: Bone marrow
OR: Overall response
RCT: Randomized controlled trial
DLI: Donor lymphocyte infusion
CR: Complete response
NR: No response
PR: Partial response
MTX: Methotrexate
mTOR: Mammalian target of rapamycin
OS: Overall survival
NRM: Non-relapse mortality
AEs: Adverse events
ITT: Intent-to-treat
mITT: Modified ITT
HR: Hazard ratios
CI: Confidence intervals
SAEs: Serious AEs
