# Supplementary material for: Mesenchymal stromal cells plus basiliximab, calcineurin inhibitor as treatment of steroid-resistant acute graft-versus-host disease: a multicenter, randomized, phase 3, open-label trial
Source: J Hematol Oncol. 2022 Mar 7;15:22. doi: 10.1186/s13045-022-01240-4 (PMC8900437; doi:10.1186/s13045-022-01240-4)

**eMethods 1. Preparation of Mesenchymal Stromal Cells**

MSCs were manufactured and provided by the Center for Stem Cell Biology and Tissue Engineering, Sun Yat-Sen University. MSCs were isolated from BM aspirates of HLA-mismatched third-party donors and cultured at a concentration of 5×10^3^ cells/m^3^ in serum-free medium. Cultures were maintained in a humidified atmosphere with 5% carbon dioxide at 37°C. After 3 days, non-adherent cells were removed and the new medium was replaced. Adherent cells were further cultured, with a change of medium every 3 days. When monolayers attained 70% to 80% confluence, cells were detached by trypsin-EDTA and passaged at a ratio of one to three. Cell products were harvested at passage 4 to 5 with each infusion product derived from a single donor. Before infusion, cells were examined for MSC characteristics and quality. Isolated cells showed positivity for CD44, CD73, CD90, CD105, and CD166, and negativity for CD34, CD45, and CD11a. The cells could be induced to differentiate into cells of the osteogenic, adipogenic, and chondrogenic lineages when cultured in appropriate induction medium and could inhibit mixed lymphocyte cultures in vitro. Multicolor fluorescence in situ hybridization showed that the cells had no chromosomal abnormalities. No infectious agents, such as bacteria, mycoplasma, or viruses, were detected in the cell supernatants or the cells themselves. No endotoxins were detected in the supernatant. Fresh meeting release criteria MSCs were shipped to the clinical sites in the 100ml saline with a continuous temperature monitoring device at 4°C. Some patients who received multiple infusions received MSCs derived from multiple donors.

**eFigure 1. Number and type of additional second-line therapy used in NR patients of the two groups.** After day 28 evaluation, 8 NR patients in the control group crossed over to the MSC group. Other second-line agents were allowed to administered to NR patients as per institutional standards in both groups, which were balanced and shown in Figure S1.


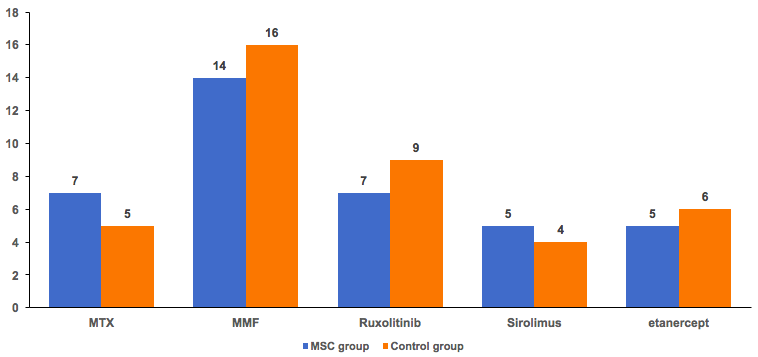

Supplement: Supplementary file 1 — Additional file 1: Method S1. Preparation of Mesenchymal Stromal Cells. [file 13045_2022_1240_MOESM1_ESM.docx]
